# Supplementary figures and images for: Complex relationship between crop yields and crop growing period: The shortened growing period before flowering contributes to yield increase in common buckwheat (Fagopyrum esculentum)
Source: PLoS One. 2025 Aug 12;20(8):e0322463. doi: 10.1371/journal.pone.0322463 (PMC12342272; doi:10.1371/journal.pone.0322463)

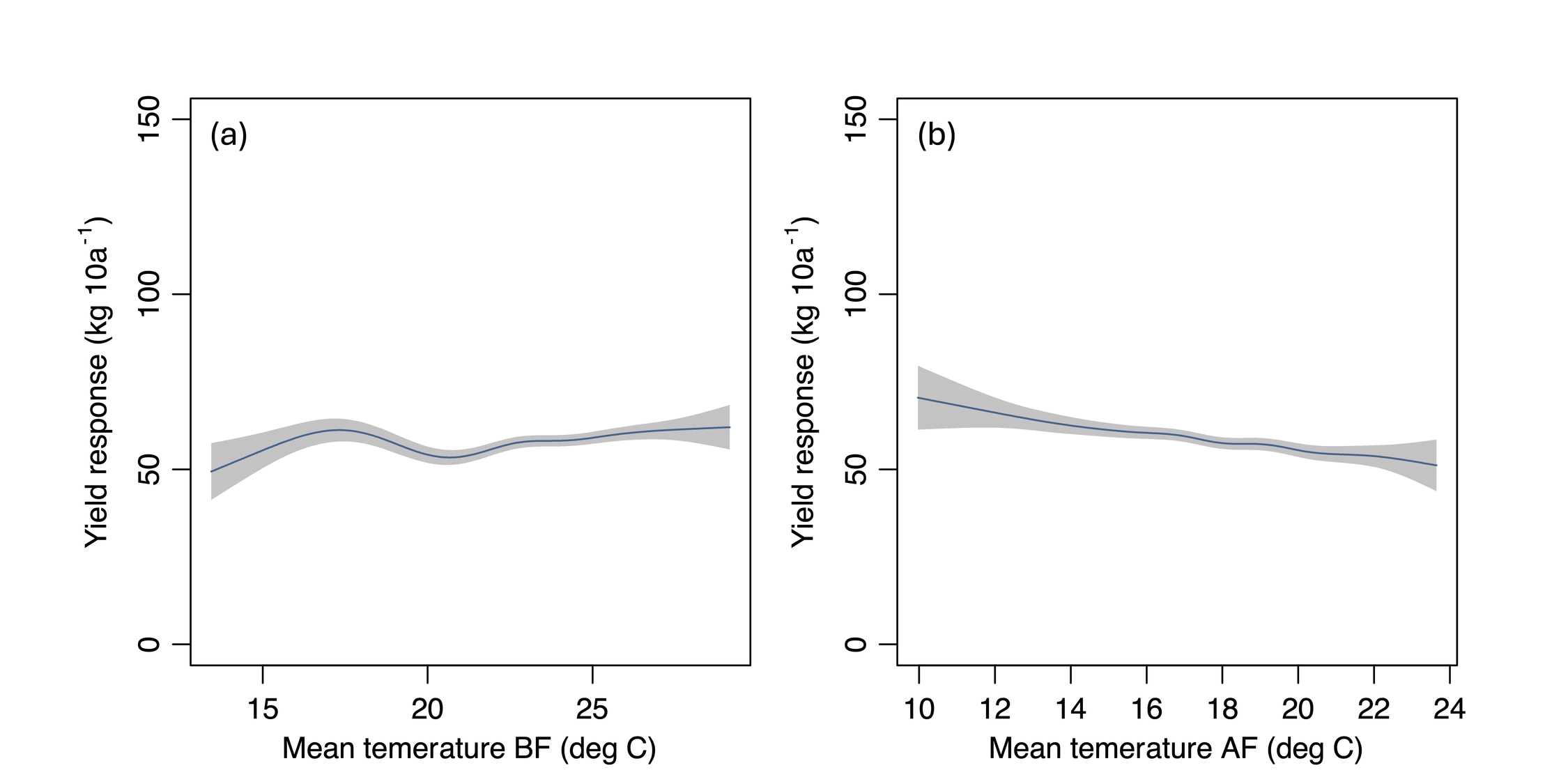

Supplement: S1 Fig — The mean temperature was averaged over the period before or after flowering. Areas shaded in gray indicate 95% confidence intervals. (TIF) [file pone.0322463.s001.tif]

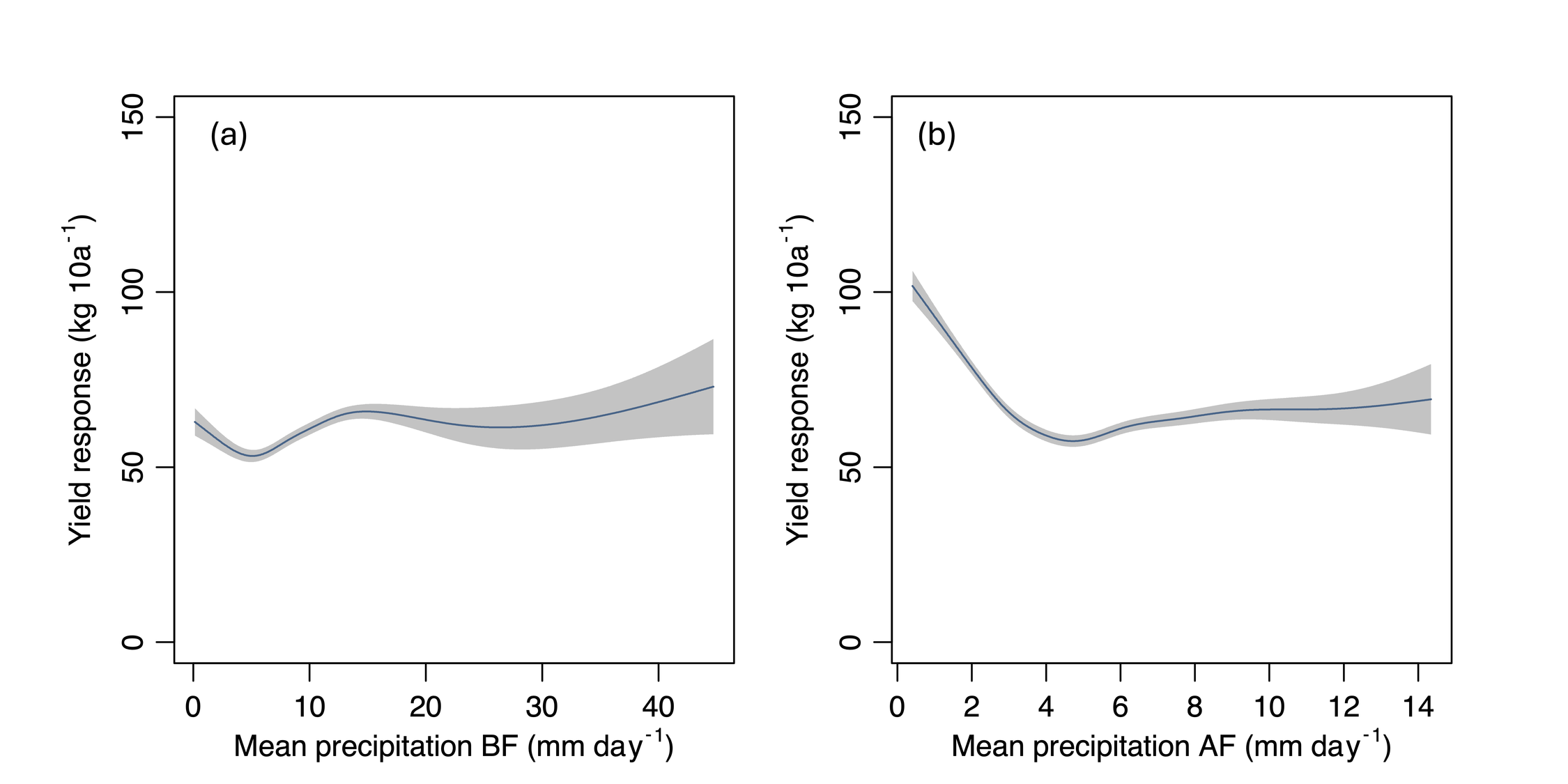

Supplement: S2 Fig — The mean precipitation was averaged over the period before or after flowering. Areas shaded in gray indicate 95% confidence intervals. (TIF) [file pone.0322463.s002.tif]

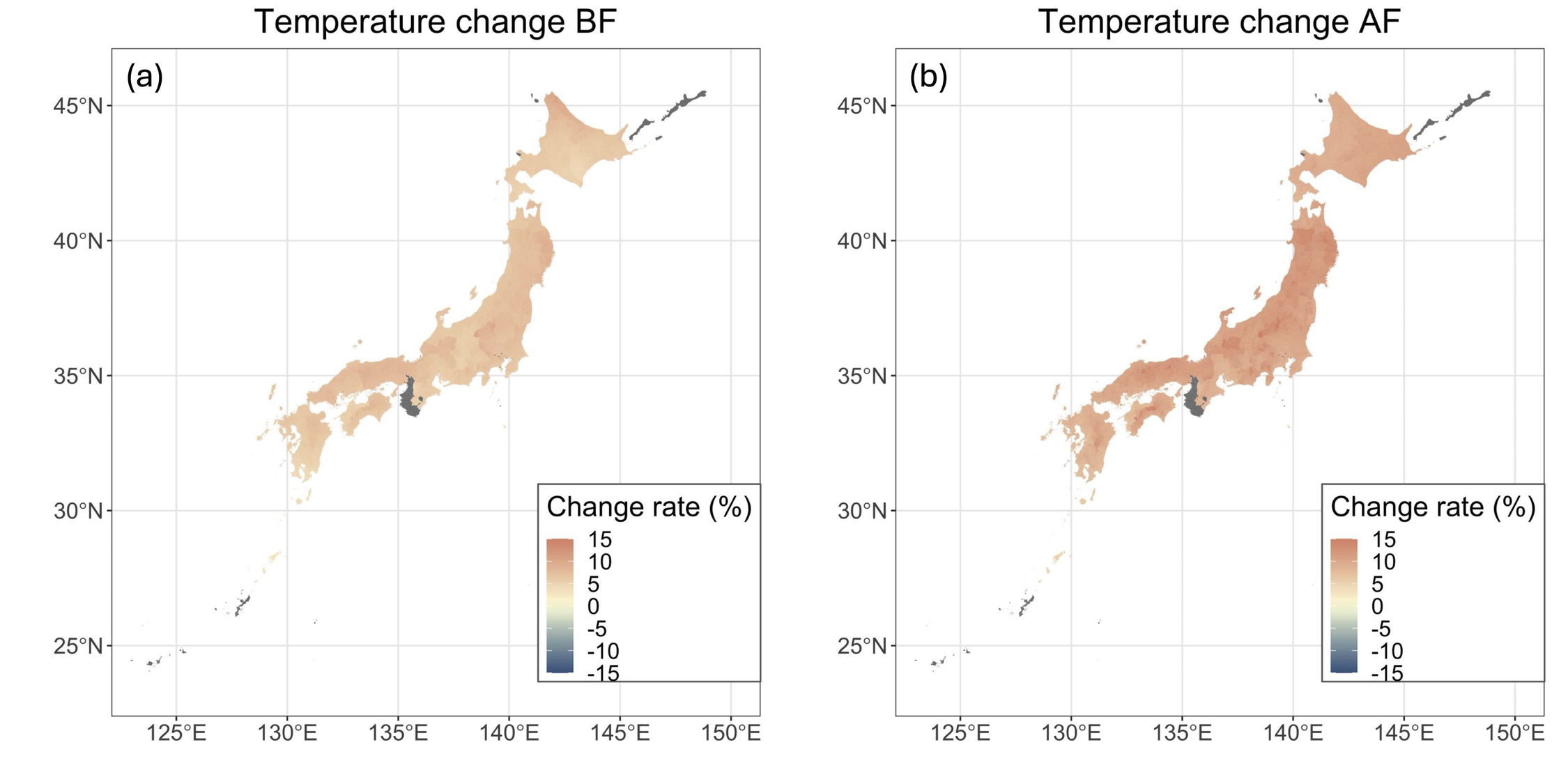

Supplement: S3 Fig — The mean values of the results predicted by the five GCMs are depicted. Areas with no data are shown in gray. The colors are divided by municipalities. The map is based on a shapefile from the National Land Numerical Information Download Site (CC BY 4.0). (TIF) [file pone.0322463.s003.tif]

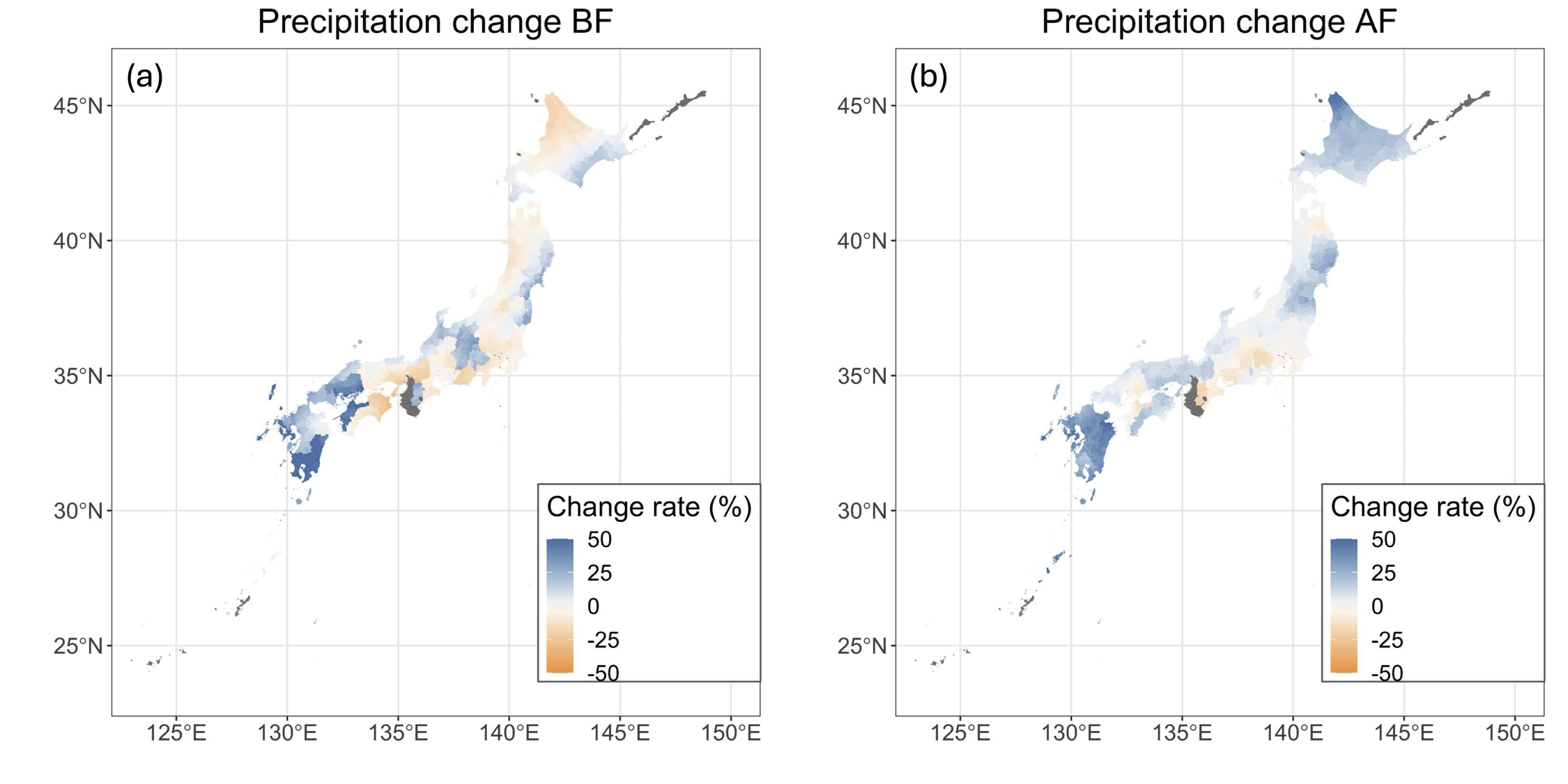

Supplement: S4 Fig — The mean values of the results predicted by the five GCMs are depicted. Areas with no data are shown in gray. The colors are divided by municipalities. The map is based on a shapefile from the National Land Numerical Information Download Site (CC BY 4.0). (TIF) [file pone.0322463.s004.tif]
