# Supplementary material for: Complex relationship between crop yields and crop growing period: The shortened growing period before flowering contributes to yield increase in common buckwheat (Fagopyrum esculentum)
Source: PLoS One. 2025 Aug 12;20(8):e0322463. doi: 10.1371/journal.pone.0322463 (PMC12342272; doi:10.1371/journal.pone.0322463)
Supplement: Attachment — Submitted filename: Response to the reviewers.pdf [file pone.0322463.s007.pdf]

Response to the editor

We would like to express our sincere gratitude to the editor for dedicating their time and expertise to providing insightful feedback on our manuscript. We deeply appreciate your careful consideration and constructive comments, which have been invaluable in improving the quality of our work. In the following, we provide our responses to the editor's comments.

It is unfortunate that we received negative comments from Reviewer 2. However, we cannot agree with Reviewer 2's comments. As we have addressed in our response to Reviewer 2, the criticisms raised represent a general indictment of all statistical analyses using crop data and stem from the reviewer's belief that experimental research is the most superior approach. In reality, experimental studies and studies employing statistical big data should be seen as complementary, not as one being superior to the other. Although statistical data inherently contain significant errors, appropriate statistical analysis can extract valuable information, and in our study we have taken the utmost care to ensure that our analyses are free from biased estimation. I would be grateful if the editor could render a fair judgment on this matter and kindly take it into consideration.

Comments: I appreciate your work on climatic predictions, especially the effect of temperature on buckwheat yield, and you have examined the solar radiation after flowering predicted to have a positive impact on yield. You have shown increased temperature with a positive impact on yield is questionable as rising temperature may cause terminal heat stress, etc. So clarification is needed on how increased temperature would certainly have yield-related benefits while not having stress-related defects on crop productivity? Hence, please add some points concerning the temperature-induced heat stress-related consequences.

Response: We sincerely appreciate your evaluation of our paper. Our paper argues that increased temperatures indirectly enhance buckwheat yield through the shortening of the pre-flowering period. In fact, as you have pointed out, our study shows that an increase in post-flowering temperature directly has a slightly negative impact on yield after flowering (S1 Fig b). However, our results indicate that rising temperatures lead to a shortened pre-flowering period, which in turn results in increased day length after flowering, thereby extending the post-flowering period, increasing the cumulative solar radiation after flowering, and ultimately enhancing yield. The fact that this yield increase outweighs the negative effects of higher post-flowering temperatures is the key factor behind the estimated yield enhancement due to increasing temperatures. As this point may have been insufficiently explained in the

original manuscript, we have revised the discussion and clarified the abstract accordingly.

In the discussion section, while describing the relationship between pre-flowering temperature and yield, we noticed that we mistakenly referred to an incorrect temperature range (LL. 321-322 and LL. 333 in the " Revised Manuscript with Track Changes"). We sincerely apologize for this error and have corrected it with the appropriate values.

Comments: Was the model you have used linear or a linear mixed model? What kind of dependent and predictor variables used for the model?

Response: The model used in this study is the Generalized Additive Model (GAM). In a GAM the relationship between explanatory variables and the dependent variable is expressed as a nonlinear function. This is explained in the Statistical Analysis section. However, for the reader's understanding, a brief explanation of the GAM has been added.

Comments: Have you assigned the fixed effects of predictor variables?

Response: All explanatory variables were assigned as fixed effects. This has been added to the Statistical Analysis section.

Comments: Please mention the average buckwheat yield per acre in 1994, 2001, and for any recent year, as you have mentioned the number of sample sizes only and not the yield.

Response: The overall average, the average for 1994 (the year with the smallest sample size), and the sample size for 2001 (the year with the largest sample size) have been added to the Past crop yield and calendar data section.

Comments: Why was the coefficient of determination ($R^2$) weakly positive with a mere 0.23 of observed and estimated yields?

Indeed, while the estimated $R^2$ is low, this is attributable to the deliberate adoption of a simple model in our statistical analysis. In our study, we utilized nationwide data from across Japan to examine the relationship between meteorological factors and yield, estimating a single spline function to represent this relationship uniformly throughout the country. This approach was intentionally chosen to address extrapolation issues in future predictions.

Although one might reasonably expect that modifying the model—for example, by estimating distinct spline functions for each municipality, as is common in many other studies—would result in a higher $R^2$, such modifications would introduce extrapolation problems when future temperature increases extend beyond the range of the training data used to construct the statistical model.

Because our model is purposefully kept simple, it inherently possesses a low degree of freedom (i.e., limited flexibility), which in turn leads to a fundamentally lower $R^2$. Nonetheless, if a statistically significant relationship between meteorological factors and yield can be detected even with such a simple model, we can confidently assert the robustness of this relationship.

Since this point is important, we have added this discussion to the final paragraph of the "The relationship between climatic factors and yield" section.

Comments: There are typos, for instance in line 57, the recent IPCC report [3]...

Response: Thank you for your comment. We have made the corrections.

Comments: Please increase the resolution of the figures.

Response: The resolution of the figures uploaded to PlosOne is high; however, it appears that the resolution is reduced when PlosOne combines the figures with the main text to create the PDF. If the resolution of the uploaded figures itself is actually low, please let me know.

Response to the reviewers.

Response to Reviewer #2:
We sincerely appreciate the reviewers' valuable comments and suggestions, which have greatly helped us improve the quality of our manuscript. Their insightful feedback has guided us in refining our analysis and presentation.

Comments: This study analyzed how buckwheat yields are affected by temperatures and day length based on long-term data from all over Japan. The results indicated that rising temperatures speed up development and increase day length in the flowering period, thus increasing yield as a result. However, because the analysis is too simple, there are various

problems and the results are unreliable.
The authors state the advantages of a simple model, but it is an oversimplification to state that the analysis without confounding factors would still produce qualitatively reliable results.
For example, buckwheat varieties are geographically unevenly distributed, which may create a spurious correlation with temperature. Also, any temporal trends in fertilizer application would affect yields, which might also show a spurious correlation with rising temperature in these decades. Furthermore, this study only deals with phenology and yield as crop data, but buckwheat plant size (biomass) is highly important. It is possible that higher temperatures could have increased plant size in a short term, resulting in higher yields. This surely have nothing to do with the effect of daylength after flowering.
If you really want to know the effects of temperature and day-length on buckwheat growth and yield, experiments under controlled environments will be able to uncover the cause-and-effect relationship, not by analyzing data that is full of uncertainties.

Answer: While it is true that our paper employs a simple statistical model, the primary objective of our study is to model the relationship between meteorological factors and both the growth rate and yield of buckwheat using historical crop data, and to discuss how buckwheat's growth rate and yield might be affected under future climate scenarios. If one were to criticize our work solely on the basis of the model's simplicity, then by the same token, every statistical study based on historical data would be rendered valueless. Moreover, experimental studies under controlled conditions are not without their own limitations. In practice, differences in fertilizer application rates, the varieties used, as well as variations in planting dates, daylength, solar radiation, and temperature, make it difficult to investigate every possible combination experimentally; indeed, very few such comprehensive studies exist. We believe that a scientifically rigorous approach should not exclude one method in favor of another; rather, combining the results of controlled experiments with statistical analyses of real-world crop data brings us closer to reliable conclusions. In fact, statistical analyses of crop yield data have already produced many valuable results in the field of climate change research, and several studies have shown that the predictive power of simple statistical models is comparable to that of process-based crop models.

We appreciate the reviewer's constructive criticism regarding the soundness of our statistical analysis from a methodological perspective. Specifically, we agree that careful consideration must be given to potential biased confounding factors, such as the geographical heterogeneity of buckwheat varieties and the temporal trends in fertilizer application, as pointed out by the reviewer.

First, although the geographical heterogeneity of varieties is not explicitly included in our model, it is implicitly incorporated. Because we employ spline functions to model the relationship between temperature and crop yield, the spline curve corresponding to each temperature range essentially represents the functional relationship between temperature and yield for the regions encompassed by that range. For example, if buckwheat exhibits low temperature sensitivity at lower latitudes and high sensitivity at higher latitudes, the spline curve for the higher temperature range would tend to be nearly flat, whereas the spline for the lower temperature range would display a pronounced relationship with temperature. Admittedly, the model does not explicitly account for fine-scale heterogeneity among varieties; however, if such heterogeneity were large—leading to markedly different responses among varieties—the estimated spline curves would tend to be parallel (i.e., indicating little response to temperature or solar radiation). Conversely, if the varieties, despite some heterogeneity, respond in roughly the same manner, the estimated spline curves would not be parallel but would instead exhibit clear slopes with respect to temperature and solar radiation. In any case, by using a simple statistical model as we have, any unbiased confounding factors will simply result in larger error terms rather than artificially imposing a relationship between meteorological factors and yield. In that sense, our analysis remains robust.

Furthermore, because temporal trends in fertilizer application could serve as a biased confounding factor, we have incorporated a spline function of year into our model. Although the amount of fertilizer applied may change from year to year, any factors that change gradually over time, independent of meteorological conditions, are absorbed by this "year" term. Consequently, any anthropogenic factors that gradually change over the years are accounted for by this component, regardless of the absolute fertilizer amounts.

It is true that statistical data inherently carry uncertainty; however, statistical methods are precisely the tools we use to extract the meteorologically relevant signals from within this random variability.

Response to Reviewer #3:

Comments: I would like to thank the authors for their effort in conducting this valuable study on buckwheat, a crucial crop with significant nutritional and agricultural benefits. Buckwheat plays a vital role in food security, especially under climate change conditions, and

understanding its phenological responses to environmental factors is essential for optimizing yield. This research provides meaningful insights into how climate variability influences buckwheat productivity, which is critical for future agricultural planning and adaptation strategies.

Answer: We sincerely appreciate the reviewer for taking the time to review our manuscript. Your thoughtful feedback has been invaluable in enhancing the quality of our work. We are honored by your evaluation and grateful for their insightful comment.
