# Supplementary material for: Complex relationship between crop yields and crop growing period: The shortened growing period before flowering contributes to yield increase in common buckwheat (Fagopyrum esculentum)
Source: PLoS One. 2025 Aug 12;20(8):e0322463. doi: 10.1371/journal.pone.0322463 (PMC12342272; doi:10.1371/journal.pone.0322463)
Supplement: Attachment — Submitted filename: Response_to_the_reviewers_auresp_3.pdf [file pone.0322463.s008.pdf]

Response to the editor

We sincerely appreciate the detailed and insightful comments provided by the editor regarding our manuscript revisions. We are deeply grateful for your thorough examination of our work and for introducing the references that have enriched our discussion. With the kind guidance of the editor, our paper has been greatly improved and refined.

Comment: Please mention the scientific name for Buck wheat (Is it a common Buck wheat or tartary
Buckwheat?) in title and at least in introduction and in required lines.

Answer: Thank you for your important comments. I have inserted the scientific name and species name into the title and added it in the main text where it was deemed necessary.

Comments: If you wish, you could check some articles for the photosynthesis efficiency and quantum yield with C3 and C4 plants:
https://ps.ueb.cas.cz/pdfs/phs/2020/01/06.pdf
https://pmc.ncbi.nlm.nih.gov/articles/PMC1066505/
I found a reference indicating the positive correlation (though not significant) between the yield and temperature in buckwheat; please check it: https://www.mdpi.com/2073-4395/11/12/2371

Response: Thank you very much for providing the valuable information on the papers, which was extremely helpful in revising the discussion section of the manuscript. These papers played a crucial role in addressing the consistency of our results. I have updated the discussion section, citing the papers you introduced (LL. 318-344 and LL. 399-403 in Revised Manuscript with Track Changes). In addition, we found an error in the paragraph of LL.318 (in Revised Manuscript with Track Changes) where "after flowering" was mistakenly written instead of "before flowering," and we have corrected it. We apologize for this oversight.
