# Supplementary material for: Complex relationship between crop yields and crop growing period: The shortened growing period before flowering contributes to yield increase in common buckwheat (Fagopyrum esculentum)
Source: PLoS One. 2025 Aug 12;20(8):e0322463. doi: 10.1371/journal.pone.0322463 (PMC12342272; doi:10.1371/journal.pone.0322463)
Supplement: Attachment — Submitted filename: Response to Reviewers.docx [file pone.0322463.s005.docx]

**Response to Reviewer**

The authors express their sincere gratitude to the reviewers for their time and effort in providing valuable feedback. Their insightful comments and suggestions have significantly contributed to enhancing the quality of this manuscript. In response to the reviewers' comments, the manuscript has been revised as follows: reviewers' comments are presented in bold italics, while the authors' responses are presented in Roman type. In the "Revised Manuscript with Track Changes" document, deletions are indicated by strikethroughs in red, and additions are indicated by blue font.

***All the libraries used in the software environment should be mentioned in the text.***

The authors appreciate the reviewers' insightful comments. In response to the reviewers' suggestions, a new section entitled "Language and Packages Used for Analysis" has been added before the Results section to provide further detail regarding the analytical tools employed in this study.

***What method was used for interpolation in the maps?***

In the figures, no interpolation method was used. The colors of the map are divided by municipalities. The total number of municipalities is more than 1,000. Therefore, while the map appears to be interpolating within the map, the colors are actually assigned to each municipality. This is also indicated in the figure legends.

***Regarding the precision and accuracy of the fitted model, statistical information (R-squared, RMSE, Index of Agreement, . . .) is needed to compare the observed values and the simulated values. Please ensure that your decision is justified on PLOS ONE’s publication criteria and not, for example, on novelty or perceived impact.***

Thank you for the reviewer's valuable feedback. We have added statistics on the accuracy between the observed and estimated values of the yield data in the Results section. We have also discussed the values of these statistics in the Discussion section.
