# Supplementary material for: Complex relationship between crop yields and crop growing period: The shortened growing period before flowering contributes to yield increase in common buckwheat (Fagopyrum esculentum)
Source: PLoS One. 2025 Aug 12;20(8):e0322463. doi: 10.1371/journal.pone.0322463 (PMC12342272; doi:10.1371/journal.pone.0322463)
Supplement: Attachment — Submitted filename: peer_review_crop.docx [file pone.0322463.s006.docx]

**Peer Review Report**

I would like to thank the authors for their effort in conducting this valuable study on buckwheat, a crucial crop with significant nutritional and agricultural benefits. Buckwheat plays a vital role in food security, especially under climate change conditions, and understanding its phenological responses to environmental factors is essential for optimizing yield. This research provides meaningful insights into how climate variability influences buckwheat productivity, which is critical for future agricultural planning and adaptation strategies.

**Title of Manuscript:**

"Complex Relationship Between Crop Yields and Crop Growing Period: The Shortened Growing Period Before Flowering Contributes to Yield Increase in Buckwheat"

**Overall Evaluation:**

This study presents an important analysis of the relationship between **crop growing period and yield**, particularly in **buckwheat under changing climate conditions in Japan**. The manuscript is well-structured and provides **valuable insights** into how **temperature variations affect crop phenology and productivity**. The use of **Generalized Additive Models (GAMs)** to assess nonlinear relationships is commendable, and the inclusion of **historical climate data and future projections (SSP5-RCP8.5)** enhances the study's relevance.

While the manuscript is **scientifically sound and methodologically robust**, there are **several areas for improvement** to strengthen the analysis, particularly regarding **statistical validation, uncertainty assessment, and the generalizability of findings beyond Japan**.

**Major Strengths:**

1. **Novelty and Scientific Contribution:**
   - The study effectively highlights the **importance of crop phase differentiation** (pre- and post-flowering) in assessing **climate change impacts on yields**.
   - It moves beyond process-based models and offers a **data-driven perspective**, which is valuable for future research and policymaking.
2. **Use of Advanced Statistical Methods:**
   - The application of **GAMs** is appropriate for capturing complex relationships between **temperature, solar radiation, and crop growth**.
   - The integration of **historical and future climate projections** adds depth to the study.
3. **Clear and Well-Presented Results:**
   - The figures and tables are well-organized and **effectively illustrate key findings**.
   - The discussion section **connects results with previous research**, adding credibility to the conclusions.

**Areas for Improvement:**

**1. Model Validation**

- The model’s predictive power (R² = 0.23) suggests it explains only a small portion of variance in crop yields. A discussion on why this level of accuracy is acceptable and how it compares to other models would improve clarity.
- Example: Compare the GAM model’s performance with a linear regression or a machine learning approach to show its relative effectiveness.

**2. Uncertainty Analysis**

- The study uses multiple climate models, but their range of predictions is not well explored. Sensitivity analysis should be included to assess how yield predictions vary under different climate assumptions.
- Example: Present confidence intervals or error margins for key results to indicate uncertainty.

**3. Generalizability of Findings**

- The study focuses exclusively on Japan, and it is unclear whether similar trends apply to other regions or crops. Expanding the discussion on broader applicability would increase the study’s impact.
- Example: Reference studies on phenological changes in other crops like wheat or rice to compare trends.

**4. Future Projections**

- The assumption that harvested area remains constant may be unrealistic, as climate change could alter land use patterns. Addressing alternative scenarios would strengthen the robustness of conclusions.
- Example: Discuss potential shifts in cultivation areas due to temperature and precipitation changes, referencing land-use models if available.

**Minor Suggestions and Editorial Improvements:**

- **Clarification of Figures:** Some figure captions (e.g., maps) could better explain **color scales and interpretations**.
- **Expansion of Literature Review:** Include studies using **alternative modeling techniques** for climate-crop interactions.
- **Terminology Consistency:** Ensure that **solar radiation effects before vs. after flowering** are clearly defined throughout.

**Additional Recommendation:**

I encourage the authors to explore studies on buckwheat stress responses in Egypt and Slovenia, particularly the work of Prof. Majeta Germ in Slovenia and Prof. Hassona in Egypt. These studies may provide further comparative insights into how different climatic and environmental conditions influence buckwheat growth and yield. Incorporating findings from diverse geographical regions can enhance the generalizability of the study.

**Final Recommendation:**

**Minor Revisions Required** -

This study provides a **valuable contribution** to the field of **climate change and crop yield dynamics**. However, improvements in **statistical validation, uncertainty assessment, and broader applicability** would enhance its robustness. Once these revisions are incorporated, the manuscript will be significantly stronger and more impactful.

I appreciate the effort and scientific rigor of the authors and encourage them to address the above suggestions to further improve the clarity and applicability of their findings.

**Confidential Comments to the Editor:**

The manuscript presents **a scientifically relevant and well-structured analysis**, but its **predictive accuracy is relatively low**, and **uncertainty assessments are limited**. I recommend **acceptance after minor to moderate revisions**, particularly **enhancing discussion on model validation, uncertainty quantification, and policy implications**.

Thanks,
